# Role of p62 in the suppression of inflammatory cytokine production by adiponectin in macrophages: Involvement of autophagy and p21/Nrf2 axis

**DOI:** 10.1038/s41598-017-00456-6

**Published:** 2017-03-24

**Authors:** Nirmala Tilija Pun, Pil-Hoon Park

**Affiliations:** College of Pharmacy, Yeungnam University, Gyeongsangbuk-do, 712-749 Republic of Korea

## Abstract

Adiponectin possesses potent anti-inflammatory properties. p62, an adaptor protein composed of multi-functional domain, is known to play a role in controlling inflammatory responses. In the present study, we examined the role of p62 in suppressing inflammatory cytokines produced by globular adiponectin (gAcrp) and the potential underlying mechanisms in macrophages. We demonstrated that gAcrp significantly increased p62 expression. Knockdown of p62 abrogated the suppressive effects of gAcrp on LPS-stimulated TNF-α and IL-1β expression and TRAF6/p38 MAPK pathway, indicating that p62 signaling is critical for suppressing inflammatory cytokines production by gAcrp. We next examined the role of p62 in gAcrp-induced autophagy activation, because autophagy has been shown to play a pivotal role in suppressing TNF-α. Herein, we observed that gene silencing of p62 prevented gAcrp-induced increases in autophagy-related genes and autophagosome formation. In addition, we found that Nrf2 knockdown prevented gAcrp-induced p62 expression, and p21 knockdown prevented Nrf2 induction, suggesting the role of p21/Nrf2 axis in gAcrp-induced p62 expression. Taken together, these findings imply that p62 signaling plays a crucial role in suppressing inflammatory cytokine production by globular adiponectin in macrophages, at least in part, through autophagy induction. Furthermore, the p21/Nrf2 signaling cascade contributes to p62 induction by globular adiponectin.

## Introduction

Adipose tissue acts as an important endocrine organ by secreting a wide range of biologically active molecules called adipokines^1^. Adiponectin is the abundant adipokine in plasma, accounting for approximately 0.01% of total plasma protein^2, 3^. Once secreted, full-length adiponectin is subjected to proteolytic cleavage and the globular domain, a cleaved form of adiponectin, circulates in the blood^4^. Although plasma levels of the globular form of adiponectin are much lower than other types of adiponectin, globular adiponectin possesses potent biological activities, including fatty acid oxidation and anti-inflammatory effects^5, 6^. Adiponectin has gained much attention recently due to its beneficial metabolic effects, in particular insulin sensitization and lipid metabolism^7–9^. Apart from its well-established metabolic role, a growing body of evidence has exhibited the anti-inflammatory properties of adiponectin. For example, adiponectin inhibited TNF-α-stimulated IL-8 synthesis in endothelial cells by modulating NF-κB signaling^10^ and normalized TNF-α production in LPS-primed Kupffer cells by suppressing ERK and p38MAPK signaling^11^. Additionally, adiponectin increased expression of anti-inflammatory mediators such as IL-10, IL-1 receptor antagonist and heme oxygenase-1^6, 12^. Although the potent anti-inflammatory properties of adiponectin have been well established, the underlying mechanisms are still largely unknown.

Autophagy is an evolutionarily conserved process of cellular degradation, in which damaged intracellular constituents are delivered by cytosolic double-membrane vesicles, known as autophagosomes, to the lysosome for degradation^13^. While autophagy was originally reported as a type of cell death distinct from apoptosis and necrosis^14, 15^, recent evidence indicates that autophagy allows for the recycling of cellular components to provide energy and building blocks in response to stressful conditions including nutrition deprivation, oxidative stress and endotoxin injury^16, 17^. Activation of autophagy leads to the degradation of TRAF6^18^ and the MYD88 adaptor protein^19^ in macrophages. In addition, liver-specific knockdown of the autophagy-related gene increased the expression of pro-inflammatory cytokines in high-fat diet-fed mice^20^, collectively indicating the prominent role of autophagy in regulating inflammation. Increasing evidence has revealed that autophagic flux is an important mechanism for various beneficial biological responses by adiponectin. For example, autophagy induction by adiponectin plays a key role in protecting liver cells from chronic ethanol consumption^21^, ameliorating high-fat diet-induced insulin resistance in skeletal muscle^22^, and developing tolerance to LPS-stimulated TNF-α production^23^.

p62, also known as p62/sequestosome-1 (p62/SQSTM1), has been described to play diverse biological roles ranging from inflammation to oxidative stress, tumorigenesis and misfolded protein degradation^24^. Recently, there is a growing appreciation that p62 is closely associated with autophagic process. p62 binds to ubiquitinated proteins through ubiquitin-associated (UBA) domain and delivers them to autophagosomes for degradation. It can also bind to LC3, an autophagosome localizing protein, through LC3 interacting region (LIR)^25^. Because LC3II is localized in the inner and outer membrane of autophagosomes, p62 is incorporated into autophagosomes and degraded^26^. Therefore, the cellular level of p62 has been considered as a marker of autophagy flux. However, there has been increasing evidence that p62 could be implicated in autophagy induction depending on cellular context and/or environments of the cells. For example, gene silencing of p62 decreases expression of autophagy-related genes and autophagosome formation in macrophages^27, 28^. Likewise, overexpression of p62 increases the basal level of autophagy by disrupting the association between Beclin-1 and Bcl-2, resulting in Beclin-1 activation^29^. Therefore, the biological role of p62 signaling in autophagic process is controversial, and it remains to be delineated which factors determine the fate of p62 in the process of autophagy.

It has been clearly demonstrated that p62 signaling is involved in a number of biological responses. In particular, p62 is well known to induce inflammatory cytokines production via TRAF6 polyubiquitination and thereby NF-κB activation^30^. Once ubiquitinated, TRAF6, together with Ubc13/Uev1A, facilitates the assembly of lysine-63 (K63)-linked polyubiquitin chain leading to the activation of IKK and subsequent NF-κB signaling^31^. In addition, p62 is involved in aPKC-mediated activation of IKK/NF-κB signaling via formation of p75-bound TRAF6 complex^32^, collectively suggesting the pro-inflammatory role of p62. In contrast to this notion, recent studies have revealed that p62 signaling is also involved in anti-inflammatory responses. For instance, p62 inhibited MYD88-TRAF6 complex formation, a vital process for activating downstream signaling cascade, to suppress expression of IL-6 and nitric oxide synthase 2 (NOS2)^33^ and p62 overexpression decreased inflammatory cytokines production^34^. Based on the previous reports, it is well established that both adiponectin and p62 signaling are implicated in autophagy induction and modulation of inflammatory responses. However, the biological role of p62 signaling in adiponectin-induced autophagy activation and anti-inflammatory responses has not been explored.

p62 cellular level is coordinately regulated via multiple mechanisms. Nuclear factor erythroid 2-related factor 2 (Nrf2), a transcription factor acting as a potent anti-oxidant regulator, has been shown to regulate p62 expression. The transcriptional activity of Nrf2 is regulated via cytosol-nucleus shuttling and Keap1 (Kelch-like ECH-associated protein 1)-mediated ubiquitination^35^. In normal condition, Keap1 binds with Nrf2 and recruits ubiquitin-proteasome factors, resulting in ubiquitination and degradation of Nrf2. However, under stress conditions, the inhibitory action of Keap1 to regulate Nrf2 is inhibited. As a result, Nrf2 translocates to the nucleus, binds to the anti-oxidant response element (ARE) and induces transcription of target genes^36^. In controlling the association between Nrf2 and Keap1, recent studies demonstrated the physiological role of p21^(CIP1/WAF1)^, an inhibitor of cyclin-dependent kinase (CDK). In addition to its typical inhibitory role in cell cycle progression, p21 has been recently shown to regulate other biological functions, including onset of cellular senescence, autophagy and inflammation^37, 38^. A recent study also indicated that p21 induction results in disruption of the interaction between Keap1 and Nrf2^39^, implying a functional role for p21 in modulating Nrf2 activation.

In the present study, to better understand the molecular mechanisms underlying anti-inflammatory properties of adiponectin, we investigated the potential role of p62 in globular adiponectin-mediated suppression of inflammatory cytokines production in macrophages. Herein, we have demonstrated that globular adiponectin induces increase in p62 expression in macrophages via p21/Nrf2 axis-dependent mechanisms. In addition, p62 induction plays a role in adiponectin-induced autophagy activation. Furthermore, we provided the first evidence that p62 signaling contributes to the suppression of LPS-stimulated inflammatory cytokine production by globular adiponectin, at least in part, via autophagy induction.

## Results

### Globular adiponectin increases p62 expression in RAW 264.7 macrophages and mouse peritoneal macrophages

**Figure 1:**
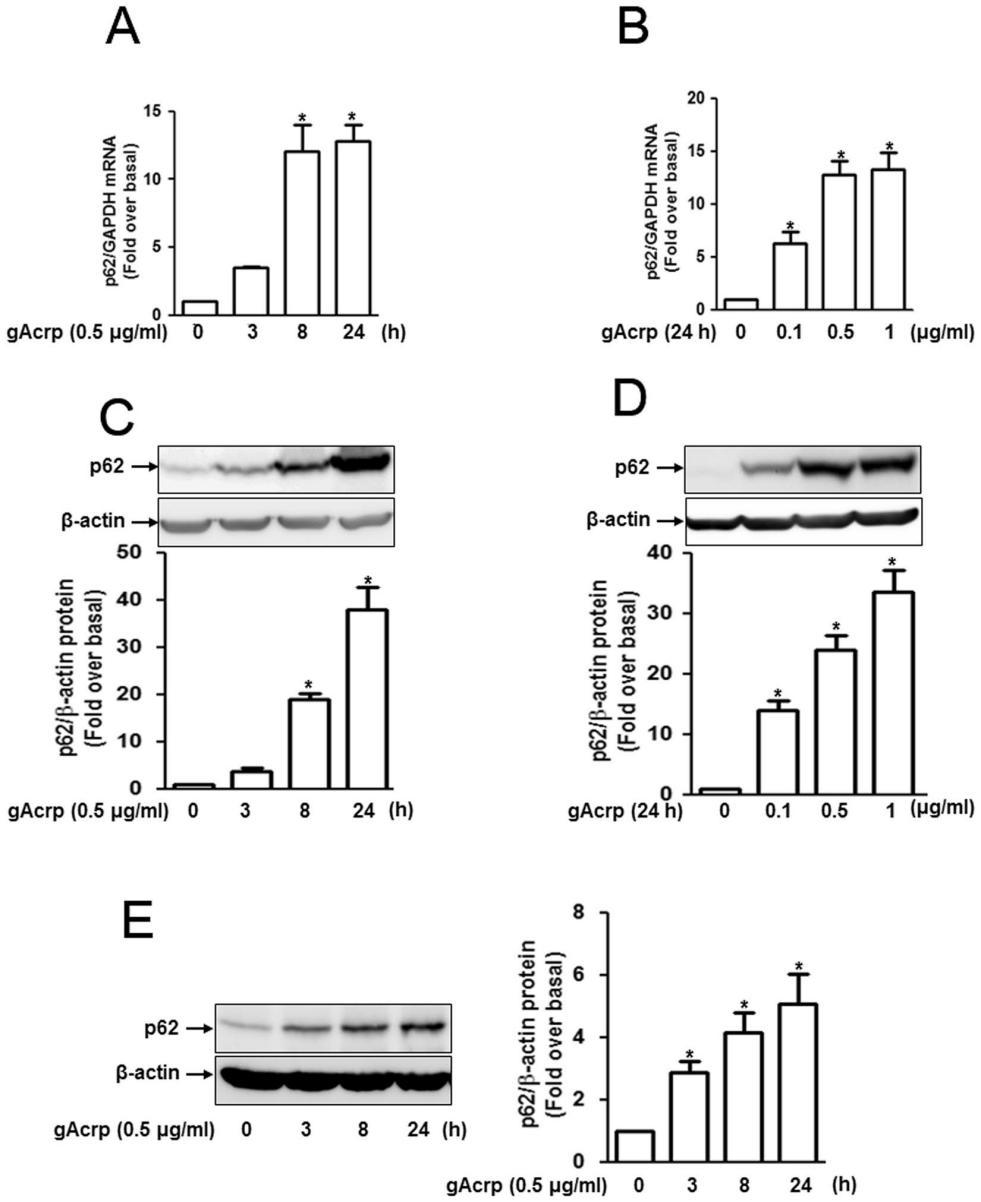
Effect of globular adiponectin on p62 expression in RAW 264.7 and murine peritoneal macrophages. (**A–D**) RAW 264.7 macrophages were stimulated with globular adiponectin (gAcrp, 0.5 μg/mL) for different time durations or different doses for 24 h. (**A** and **B**) p62 mRNA levels were determined by quantitative RT-PCR analysis and normalized to housekeeping gene GAPDH mRNA. Values represent fold change relative to the unstimulated control cells and are presented as mean ± SEM (n = 3). *P < 0.05 indicates the comparison with control cells (**C** and **D**) Protein expression levels of p62 were measured by Western blot analysis as indicated in materials and methods. Representative images from three sets of separate experiments are presented along with β-actin as an internal loading control. (**E**) Macrophages were isolated from peritoneum of mice and treated with gAcrp (0.5 μg/mL) for the indicated time periods. p62 protein expression levels were determined by Western blot analysis. Representative images are shown along with β-actin as an internal loading control. Quantitative analysis of p62 protein expression was performed by densitometric analysis and shown in the lower (or right) panel (**C–E**). Values presented are fold change compared to control cells and expressed represented as mean ± SEM (n = 3). *P < 0.05 compared to the unstimulated control cells.

To explore the role of p62 in anti-inflammatory responses of adiponectin, we first observed the effect of globular adiponectin (gAcrp) on p62 expression. As indicated in Fig. 1, treatment of RAW 264.7 macrophages with gAcrp significantly increased p62 mRNA (Fig. 1A and B) and protein expression (Fig. 1C and D) in time- and dose-dependent manner. Effect of adiponectin on p62 expression was further confirmed in primary peritoneal macrophages as indicated in Fig. 1E. These results collectively indicate that globular adiponectin enhances p62 expression in macrophages.

### Globular adiponectin increases p62 expression in a paracrine manner and adipoR2 receptor plays a crucial role in p62 expression in macrophages

**Figure 2:**
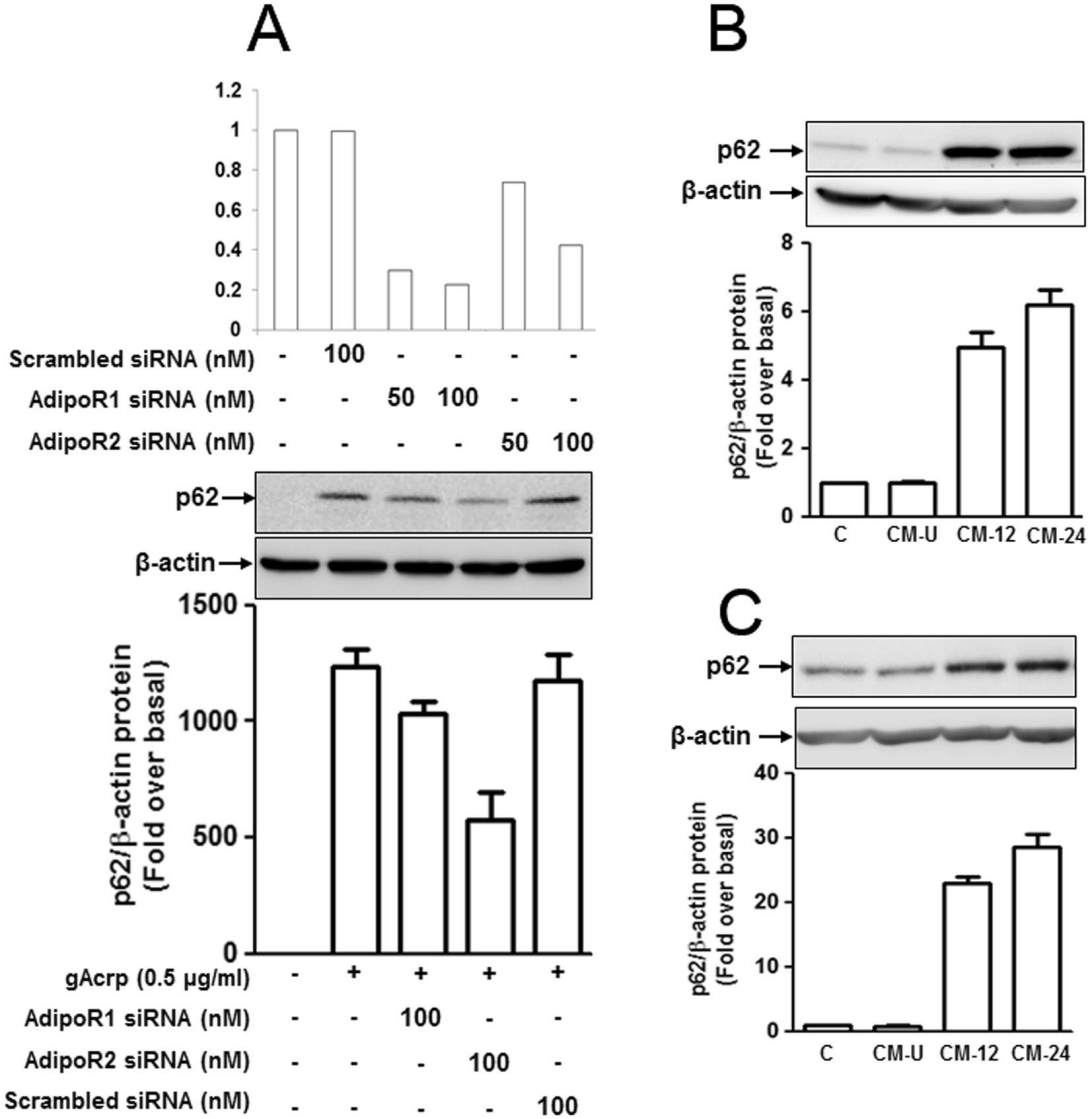
Critical role of adiponectin receptor type 2 and the paracrine effect of globular adiponectin on p62 induction in macrophages. (**A**) RAW 264.7 macrophages were transfected with siRNA targeting either adiponectin receptor type 1 (adipoR1), adiponectin receptor type 2 (adipoR2) or scrambled control siRNA for 24 h, followed by stimulation with gAcrp (0.5 μg/mL) for 24 h. (Upper panel) Transfection efficiency of siRNA was measured by qRT-PCR after 24 h of transfection. (Lower panel) Protein expression level of p62 was detected by Western blot analysis. Representative images from the three independent experiments are shown along with β-actin as an internal loading control. (**B** and **C**) RAW 264.7 macrophages (**B**) or murine peritoneal macrophages (**C**) were stimulated with adiponectin and conditioned media (CM) were prepared as indicated in materials and methods. Cells were then stimulated with conditioned medium (CM) or unstimulated conditioned medium (CM-U), prepared from the cells not stimulated with gAcrp. Western blot analysis was performed to detect p62 protein expression. Images are representative of three separate experiments that showed similar results. p62 protein expression was quantitated by densitometric analysis and shown in the below panel. Values represented the fold change relative to control cells (fold over basal) and are presented as mean ± SEM (n = 3). *P < 0.05 as compared to the control cells; ^#^P < 0.05 as compared to globular adiponectin treated cells, but not transfected with adiponectin receptor siRNA.

The biological activities of adiponectin occur upon binding with its receptors, adiponectin receptor type 1 (adipoR1) and type 2 (adipoR2)^40^. To identify the main receptor type involved in gAcrp-induced p62 expression, RAW 264.7 macrophages were transfected with siRNA targeting adipoR1 or adipoR2. As shown in Fig. 2A, p62 induction by gAcrp was markedly attenuated by gene silencing of adipoR2, while only slight reduction was observed by knocking down of adipoR1, suggesting that adipoR2 signaling, rather than adipoR1, plays an essential role in adiponectin-induced p62 expression.

Diverse biological responses by adiponectin are mediated by various secreted mediators^23, 41^, demonstrating a paracrine mechanism of action. To determine the paracrine effect of adiponectin for p62 expression, we prepared conditioned media (CM) and analyzed its effects on p62 expression. As shown in Fig. 2, stimulating RAW 264.7 macrophages (Fig. 2B) or peritoneal macrophages (Fig. 2C) with CM resulted in a significant increase in p62 expression similar to the effects observed by treatment with gAcrp, whereas no significant effect was observed by unstimulated conditioned media (CM-U), implying a paracrine effect of adiponectin on p62 expression.

### p62 expression plays a key role in the suppression of LPS-stimulated inflammatory cytokines production by globular adiponectin in macrophages

**Figure 3:**
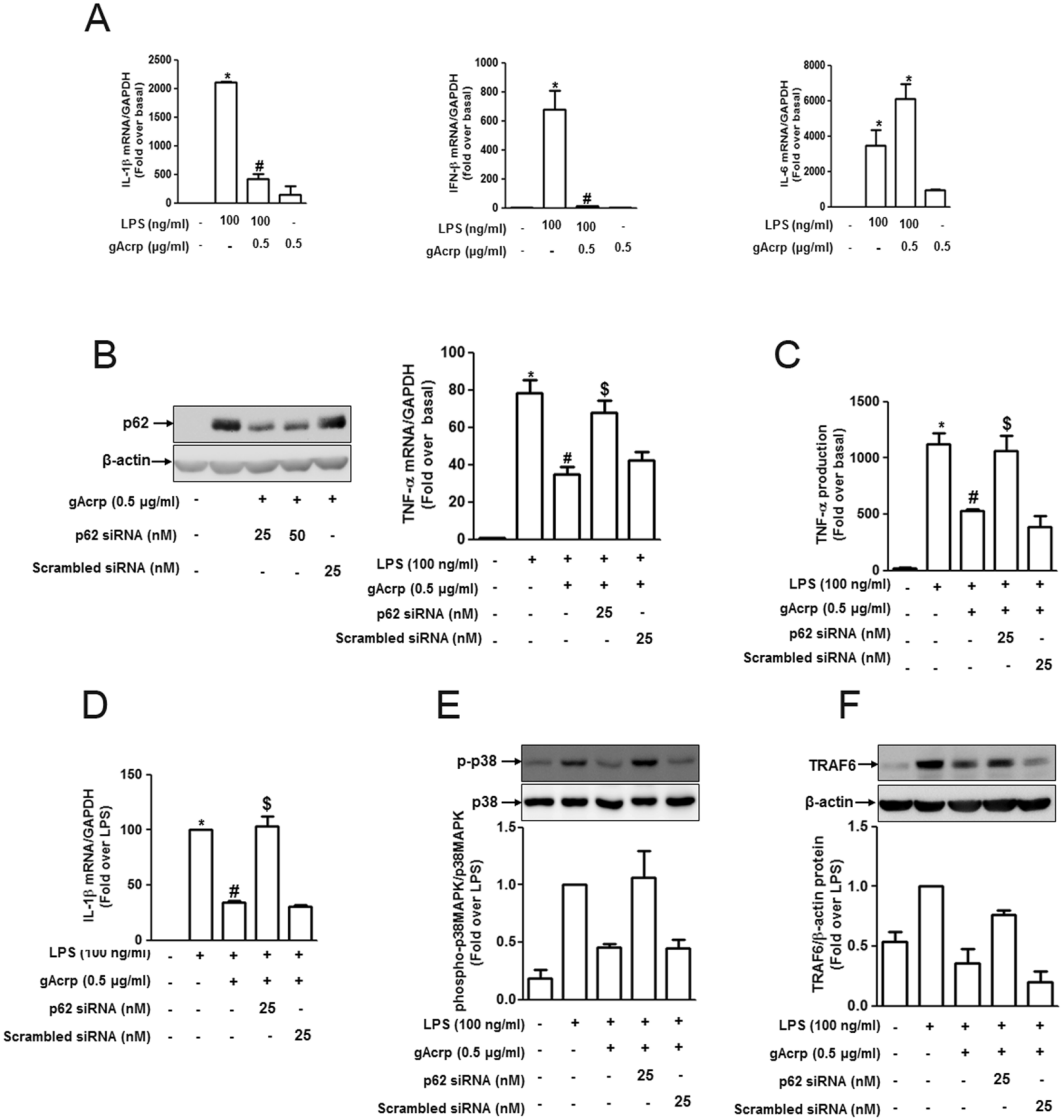
Involvement of p62 induction in the suppression of LPS-induced inflammatory cytokine production by globular adiponectin in RAW 264.7 macrophages. (**A**) Cells were pretreated with gAcrp (0.5 μg/mL) for 24 h followed by incubation with LPS (100 ng/mL) for additional 6 h for the measurement of IL-1β, IFN-β and IL-6. Messenger RNA expression levels of these pro-inflammatory cytokines were measured by qRT-PCR and normalized to the level of GAPDH mRNA. (**B**) RAW 264.7 macrophages were transiently transfected either with p62 siRNA or scrambled siRNA. Cells were then pretreated with gAcrp (0.5 μg/mL) for 24 h followed by stimulation with LPS (100 ng/mL) for an additional 2 h. (Left panel) Transfection efficiency of p62 siRNA was monitored by Western blot analysis 24 h after transfection. (Right panel) TNF-α mRNA level was detected by qRT-PCR analysis. (**C**) After transfection with p62 siRNA, RAW 264.7 macrophages were incubated with gAcrp followed by LPS (100 ng/mL) treatment for 4 h. The supernatants were then collected and used for measurement of secreted TNF-α by ELISA as described in methods. (**D**) After transfection with p62 siRNA and pretreatment with gAcrp, RAW 264.7 macrophages were stimulated with LPS (100 ng/mL) for an additional 6 h. Total RNA was isolated, reverse transcribed to cDNA and used for PCR amplification to measure IL-1β mRNA level was measured by qRT-PCR. (**E** and **F**) RAW 264.7 macrophages were transfected either with p62 siRNA or scrambled siRNA for 24 h. Cells were then pretreated with adiponectin (0.5 μg/mL) for 24 h followed by stimulation with LPS (100 ng/mL) for additional 30 min. Phosphorylation of p38MAPK (**E**) and TRAF6 expression (**F**) were evaluated by Western blot analysis. Images are representative of three independent experiments. Quantitative analyses of phosphorylated p38MAPK (**E**) and TRAF6 expression (**F**) shown in the lower panels were performed by densitometric analysis. Values are presented as mean ± SEM (n = 3). *P < 0.05 compared to the control cells; ^#^P < 0.05 as compared to the cells treated with LPS; ^$^P < 0.05 compared to cells treated with LPS and adiponectin, but not transfected with p62 siRNA.

Next, we examined the functional role of p62 in suppressing inflammatory cytokines production by adiponectin. For this, we first confirmed suppressive effects of adiponectin on inflammatory cytokines expressions in our experimental conditions. We observed that gAcrp significantly suppressed LPS-induced TNF-α expression^23^ (Supplementary Figure 1), and LPS-stimulated IL-1β and IFN-β expression consistent with previous observations^23, 42^, while no inhibitory effect on IL-6 expression was observed (Fig. 3A).

To verify the functional role of p62 signaling in suppressing inflammatory cytokines production, cells were transfected with siRNA targeting p62. As indicated in Fig. 3, knockdown of p62 abrogated suppression of TNF-α both in mRNA and protein (Fig. 3B and C, respectively), and IL-1β mRNA expression (Fig. 3D) with no effect on IFN-β (Supplementary Figure 2), implying that p62 induction plays a role in suppressing LPS-induced inflammatory cytokines production in a gene-selective manner.

In order to disclose the molecular mechanisms underlying p62-mediated suppression of inflammatory cytokine production, we performed additional experiments to determine the role of p62 signaling in the modulation of TRAF6 and p38 MAPK, a key signaling mechanism involved in LPS-stimulated inflammatory cytokine production^43^. We observed that suppression of p38 MAPK phosphorylation (Fig. 3E) and TRAF6 expression (Fig. 3F) by gAcrp was abolished by p62 siRNA transfection. Taken together, these results suggest that p62 induction plays a critical role in the suppression of inflammatory cytokines expression by adiponectin, probably via inhibition of the TRAF6/p38 MAPK pathway. To assure the proper use of gAcrp without significant effect by endotoxin contamination, as it is prepared by processing *E. coli*, we treated the cells with polymyxin B (PMB), which inhibits LPS binding protein and inactivates LPS signaling, and found that polymyxin B significantly reduced LPS-stimulated TRAF6 expression and ERK phosphorylation, while no significant effects were observed in gAcrp-induced suppression of TRAF6 expression and ERK phosphorylation (Supplementary Figure 3A and B), excluding the possibility that endotoxin contamination may contribute to gAcrp-induced various biological responses observed in this study.

### p62 induction mediates expression of autophagy-related genes and autophagosome formation by globular adiponectin in RAW 264.7 macrophages

**Figure 4:**
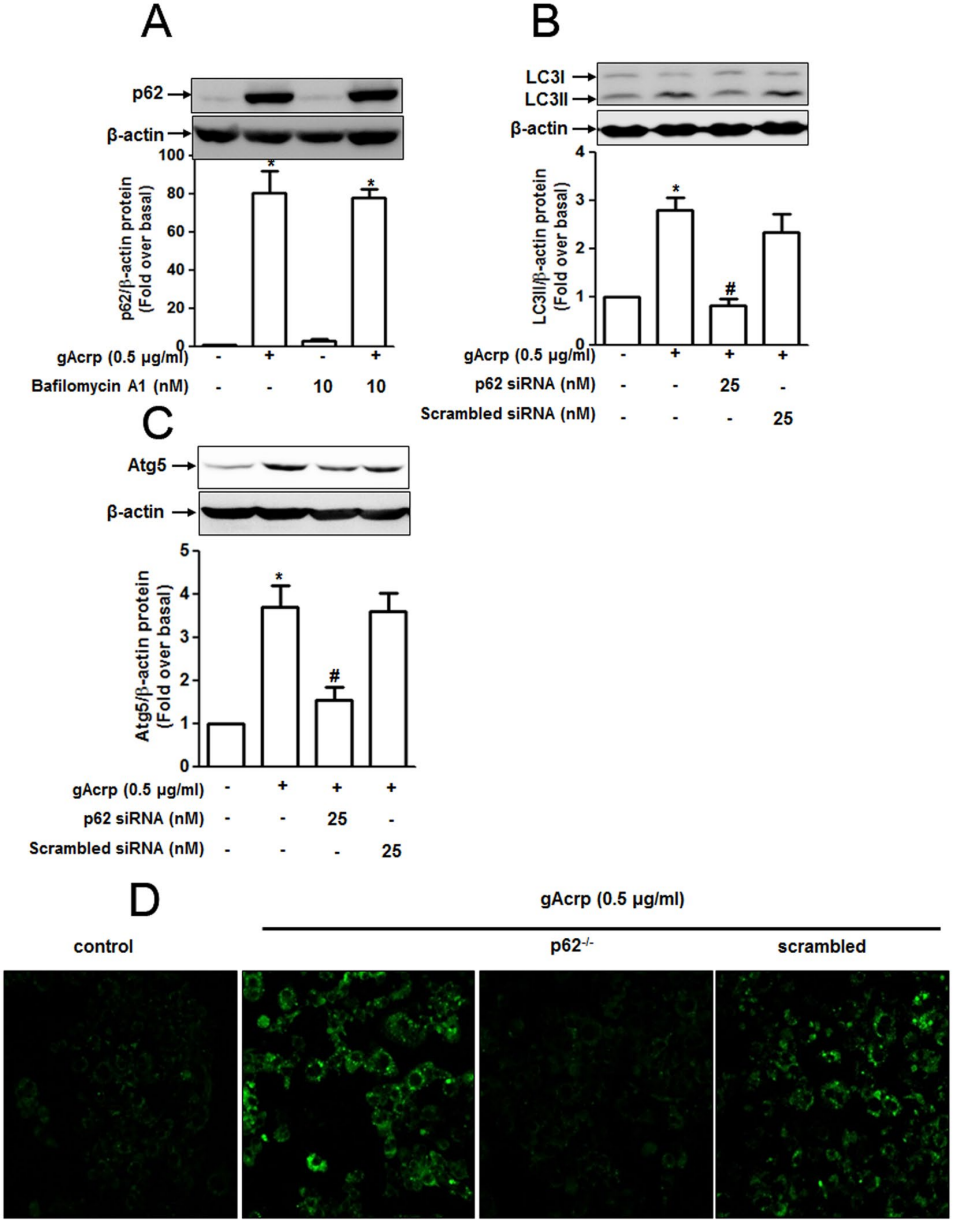
p62-mediated autophagy induction is implicated in the modulation of TRAF/p38 MAPK signaling pathway. (**A**) Cells were pretreated with bafilomycin A1 for 2 h followed by gAcrp (0.5 μg/mL) treatment for an additional 24 h. The p62 protein expression was detected by Western blot analysis. Images are representative of three separate. β-actin was taken as an internal loading control. (**B** and **C**) Cells were transfected either with p62 siRNA or scrambled siRNA for 24 h, followed by adiponectin treatment for 24 h (for the measurement of LC3 accumulation) or for 8 h (for the measurement of Atg5 expression). Accumulation of LC3 II (B) and expression of Atg5 (**C**) were measured by Western blot analysis. Quantitative analyses of p62 (**A**), LC3 II (**B**) and Atg5 (**C**) shown in the below panels were performed by densitometric analysis. Values are shown as mean ± SEM (n = 3). *P < 0.05 compared with control cells; ^#^P < 0.05 compared to with adiponectin treated cells. (**D**) After transfection with p62 siRNA, cells were transfected with GFP-tagged LC3 plasmid for 48 h followed by adiponectin treatment for 24 h. GFP-LC3 puncta are indicated with white arrows and images were captured with an A1 Confocal imaging System. Representative images that showed similar results from three independent experiments are shown.

We next investigated the involvement of p62 signaling in gAcrp-induced autophagy activation in our experimental condition. For this, we first examined whether autophagy flux causes p62 protein degradation in adiponectin-treated macrophages. As shown in Fig. 4A, treatment with Bafilomycin A1, a pharmacological inhibitor of autophagy flux, did not further enhance gAcrp-induced increase in p62 expression, suggesting that p62 would not be a target of autophagy flux in this condition. We next determined if p62 induction is implicated in gAcrp-induced autophagy activation. As depicted in Fig. 4, gene silencing of p62 significantly suppressed gAcrp-induced LC3 II accumulation (Fig. 4B), Atg5 expression (Fig. 4C) and autophagosome formation (indicated by LC3 puncta) (Fig. 4D), implying that p62 signaling has an inducing effect in autophagy activation by gAcrp. In this study, the experiments have been conducted at lengthy duration (24 h) of gAcrp stimulation. To determine whether LC3II accumulation is due to autophagy induction or blockade of autophagy, we measured expression level of LC3II by gAcrp at different time durations in the absence or presence of Bafilomycin. Globular adiponectin rapidly induced LC3II accumulation as early as 1 h, which are sustained up to 24 h (Supplementary Figure 8), suggesting the autophagy inducing effect by gAcrp. In addition, adiponectin-induced LC3II accumulation was further enhanced by Bafilomycin, indicating that gAcrp induces autophagy flux. These results collectively suggest that gAcrp treatment causes both autophagy induction and autophagy flux, but LC3II accumulation would not be due to the blockade of autophagy.

We have previously shown that autophagy induction contributes to the development of a tolerance to LPS-stimulated TNF-α expression through modulation of p38MAPK/TRAF6 axis^23^. Herein, we also observed that LC3B knockdown abrogated suppression of p38 MAPK phosphorylation and TRAF6 expression (Supplementary Figure 4A and B) in this experimental condition, confirming a critical role of autophagy in the suppression of inflammatory cytokine production by globular adiponectin through modulating the TRAF6/p38 MAPK pathway. These data exactly correlate with the results obtained from gene silencing of p62 (Fig. 3E and F), suggesting that p62 signaling plays a crucial role in the suppression of LPS-stimulated inflammatory cytokine expression by gAcrp via autophagy induction.

### Nrf2 signalling is involved in p62 expression and autophagy induction by globular adiponectin in RAW 264.7 macrophages

**Figure 5:**
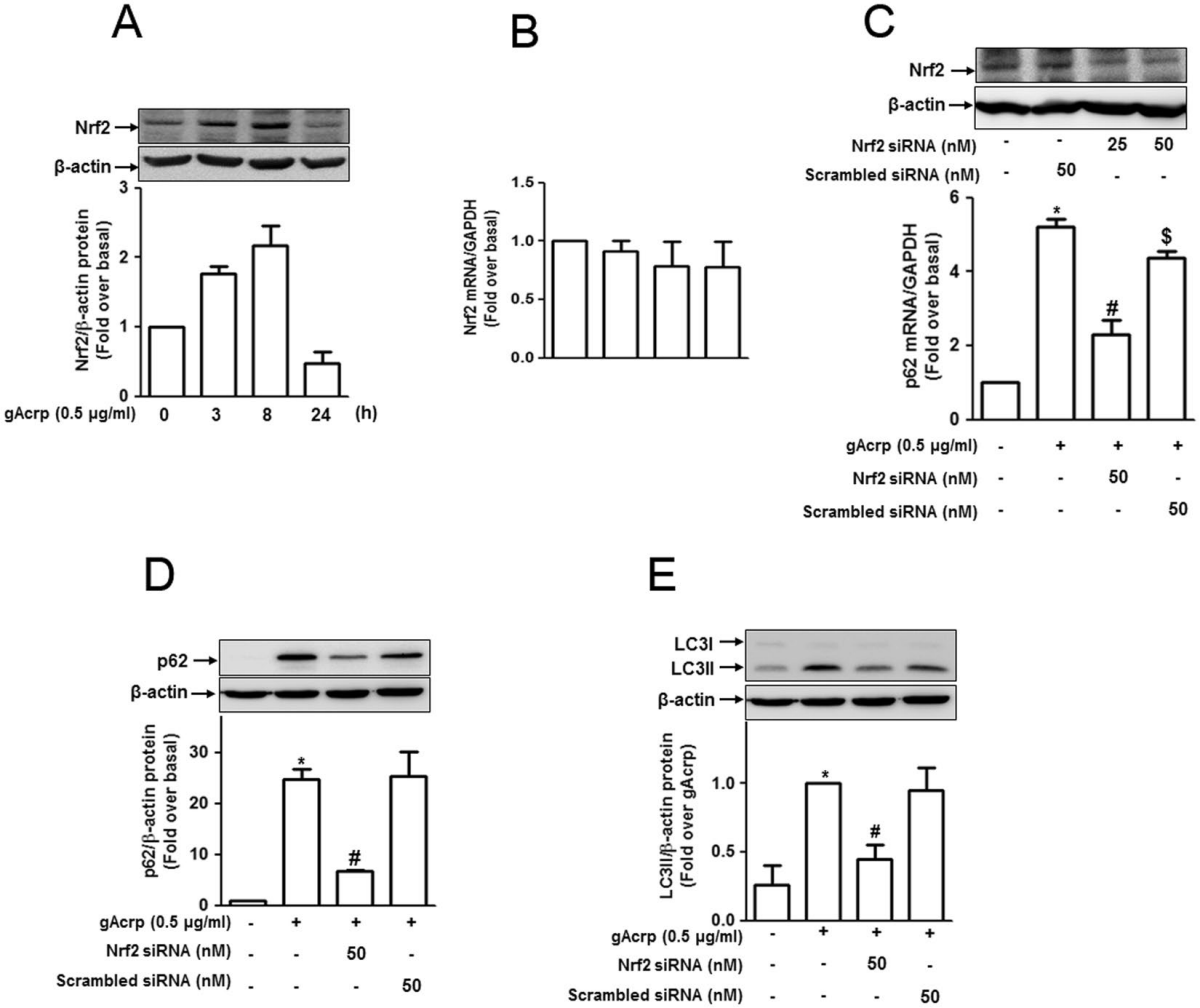
Nrf2 is required for the globular adiponectin-mediated upregulation of p62 and LC3 II accumulation in RAW 264.7 macrophages. (**A** and **B**) Cells were treated with adiponectin (0.5 μg/mL) for the indicated time periods. (**A**) Total Nrf2 protein expression level was determined by Western blot analysis. Representative images of three separate experiments are shown along with β-actin as an internal loading control. (**B**) Nrf2 mRNA expression was determined by qRT-PCR and normalized to GAPDH mRNA levels. Values represent fold change relative to the control cells and are presented as mean ± SEM (n = 3). (**C–E**) RAW 264.7 macrophages were transfected either with Nrf2 siRNA or scrambled siRNA for 24 h followed by treatment with adiponectin (0.5 μg/mL) for 24 h. (**C**) (Upper panel) Transfection efficiency of Nrf2 siRNA was determined by Western blot analysis after 24 h of transfection. (Lower panel) Messenger RNA level of p62 was measured by qRT-PCR. (**D**) Protein expression level of p62 was measured by Western blot analysis. Representative images of three independent experiments are shown along with β-actin. (**E**) Accumulation of LC3 II was detected by Western blot analysis as described previously. Quantitative analyses of Nrf2 (**A**), p62 (**D**) protein expression, and accumulation of LC3 II (**E**) shown in the lower panels were performed by densitometric analysis. Values are expressed as mean ± SEM (n = 3). *P < 0.05 compared to control cells; ^#^P < 0.05 compared to untransfected cells treated with globular adiponectin.

To further elucidate relevant signaling mechanisms, we examined whether Nrf2 signaling contributes to gAcrp-induced p62 expression. We first assessed the effect of gAcrp on Nrf2 expression and observed that gAcrp treatment rapidly increased Nrf2 protein (Fig. 5A) without no significant effect on mRNA level (Fig. 5B), indicating the post-transcriptional regulation of Nrf2 by gAcrp. Next, to examine the functional role of Nrf2 signaling in p62 expression, cells were transfected with Nrf2 siRNA, and p62 expression was investigated. Nrf2 knockdown significantly decreased gAcrp-induced p62 mRNA and protein expression (Fig. 5C and D), implying a crucial role for Nrf2 signaling in gAcrp-induced p62 expression. Furthermore, Nrf2 knockdown significantly inhibited gAcrp-induced LC3II accumulation (Fig. 5E), consistent with the results obtained from p62 knockdown (Fig. 4B), indicating the role of Nrf2 signaling in gAcrp-induced autophagy activation. Taken together, these results imply that adiponectin-induced Nrf2 accumulation is involved in transcriptional activation of p62, which subsequently induces autophagy in macrophages.

### p21 induction contributes to globular adiponectin-induced Nrf2 expression by modulating ubiquitination in RAW 264.7 macrophages

**Figure 6:**
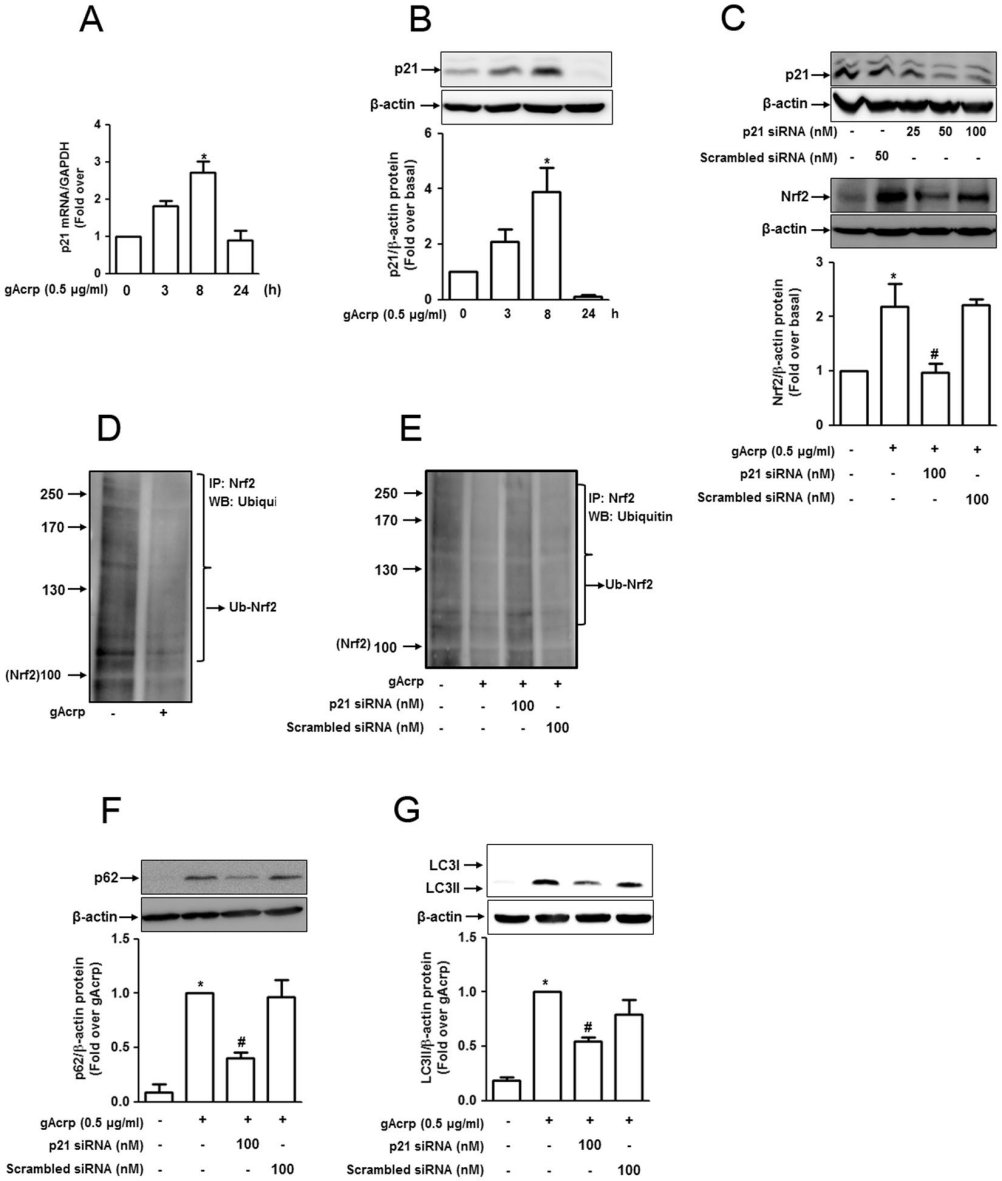
p21 induction plays a role in globular adiponectin-induced upregulation of Nrf2 and p62 in RAW 264.7 macrophages. (**A** and **B**) RAW 264.7 macrophages were stimulated with globular adiponectin (0.5 μg/mL) for different time durations. (**A**) p21 mRNA levels were measured by qRT-PCR analysis and normalized to mRNA of GAPDH. Values represent fold change relative to the control cells and are presented as mean ± SEM (n = 3). ^*^P < 0.05 compared with control cells. (**B**) Protein expression levels of p21 were analyzed by Western blot analysis. (**C**) RAW 264.7 macrophages were transfected either with p21siRNA or scrambled siRNA followed by treatment with adiponectin for 6 h. (Upper panel) Transfection efficiency of p21 siRNA was monitored by Western blot analysis after 24 h of transfection. (Lower panel) Nrf2 protein level was determined by Western blot analysis. (**D**) Cells were treated with globular adiponectin (0.5 μg/mL) for 6 h and the ubiquitinated Nrf2 level was determined by Immunoprecipitation using an anti-Nrf2 antibody and further immunoblotting with anti-ubiquitin antibody. (**E**) Cells were transfected with p21 siRNA or scrambled siRNA, followed by treatment with globular adiponectin for additional 6 h. Nrf2 protein ubiquitination was determined by Immunoprecipitation assay in a manner similar to panel D. Representative images of three separate experiments are shown. (**F** and **G**) p21 gene was silenced by transfection with siRNA targeting p21 or scrambled control siRNA. Cells were then treated with globular adiponectin (0.5 μg/mL) for 24 h. Expression of p62 (**F**) and accumulation of LC3 II (**G**) were analyzed by Western blot analysis. Representative images of three separate experiments are shown along with β-actin as an internal control. Quantitative analyses for protein expression level of p21 (**B**), Nrf2 (**C**), p62 (**F**) and accumulation of LC3 II (**G**) shown in lower panels were performed by densitometric analysis. Values are expressed as mean ± SEM (n = 3) *P < 0.05 compared to control; ^#^P < 0.05 compared to the cells treated with globular adiponectin, but not transfected with p21 siRNA.

To further determine the upstream signaling pathways implicated in Nrf2 expression, we investigated the role of p21 signaling, which has been shown to stabilize Nrf2 via regulation of ubiquitination^39^, in agreement with our previous observations demonstrating the post-transcriptional regulation of Nrf2 by gAcrp. As indicated in Fig. 6, gAcrp rapidly increased p21 mRNA expression and returned to the normal level at 2 h treatment (Fig. 6A) in a pattern similar to the protein expression (Fig. 6B). In addition, p21 gene silencing significantly inhibited gAcrp-induced Nrf2 expression (Fig. 6C). To further clarify the mechanisms underlying p21-mediated Nrf2 expression by gAcrp, we hypothesized that p21 directly interacts with Nrf2 and modulates its ubiquitination. In continuing experiments, we observed that globular adiponectin significantly decreased ubiquitinated level of Nrf2 (Fig. 6D). Moreover, gene silencing of p21 restored decrease in Nrf2 ubiquitination (Fig. 6E), suggesting that p21 induction by gAcrp protects Nrf2 from ubiquitination-mediated degradation. Additionally, p21 knockdown significantly inhibited gAcrp-induced expression of p62 (Fig. 6F) and LC3II (Fig. 6G), confirming that the p21/Nrf2 pathway acts as the upstream signaling of p62 expression, thereby contributing to autophagy activation by gAcrp.

### Adiponectin receptor type 2 (AdipoR2) predominantly modulates signaling events underlying globular adiponectin-induced autophagy in RAW 264.7 macrophages

**Figure 7:**
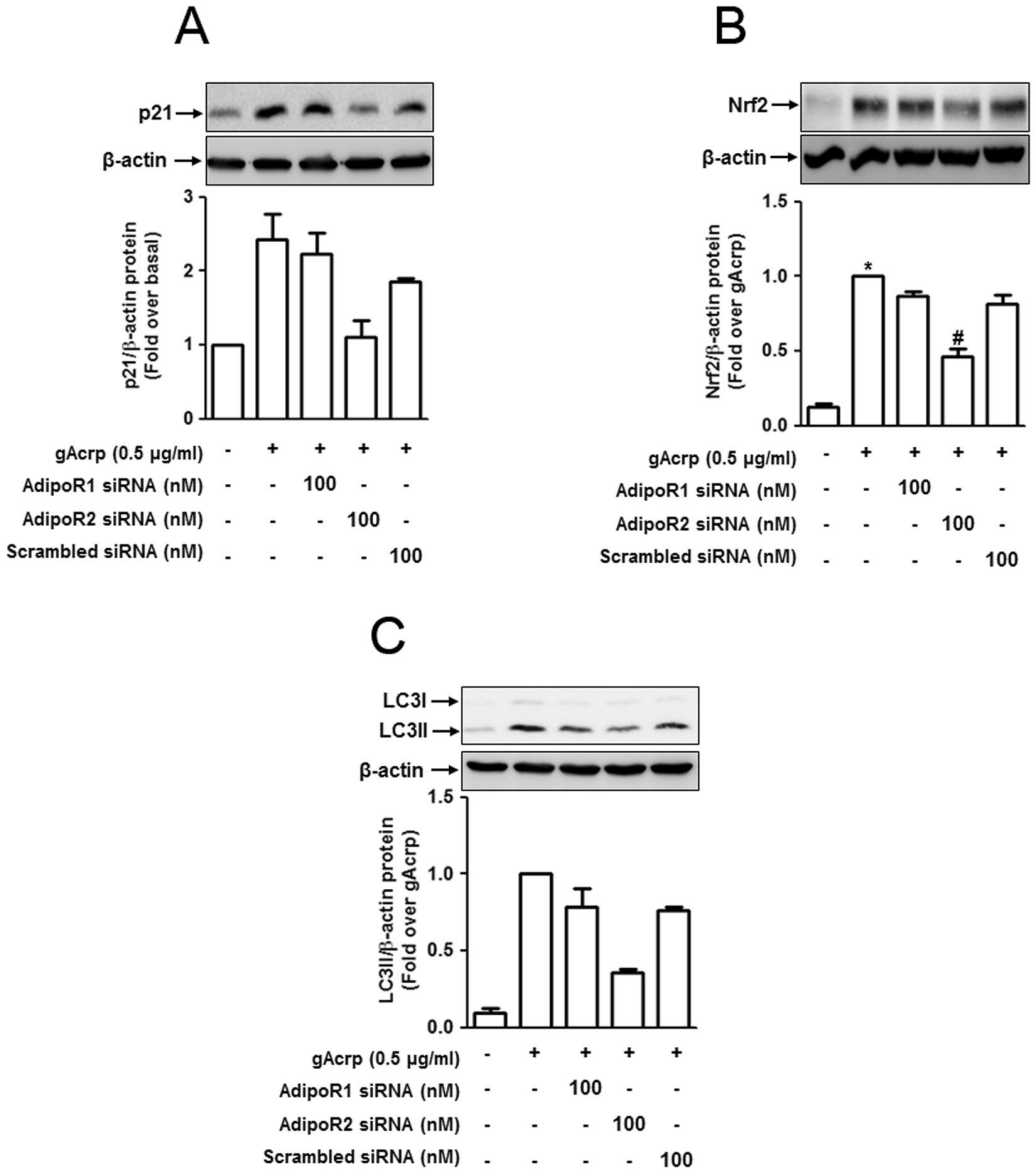
Adiponectin receptor type 2 plays a key role in globular adiponectin-induced p21 and Nrf2 expression, and accumulation of LC3 II in RAW 264.7 macrophages. RAW 264.7 macrophages were transfected with an siRNA targeting AdipoR1, AdipoR2 or scrambled control siRNA for 24 h followed by treatment with adiponectin (0.5 μg/mL) for 6 h (for measuring p21 and Nrf2 expression) or for 24 h (for LC3 II). The expression level of p21 (**A**) and Nrf2 (**B**), and accumulation of LC3 II (**C**) were measured by Western blot analysis. The representative of three separate experiments has been presented along with β-actin as an internal control. Quantitative analyses of p21 (**A**), Nrf2 (**B**), and accumulation of LC3 II (**C**) shown in lower panels were performed by densitometric analysis. Values are shown as mean ± SEM (n = 3). *P < 0.05 compared to the control groups; ^#^P < 0.05 compared to untransfected cells treated with globular adiponectin.

As adipoR2 plays a crucial role in gAcrp-induced p62 expression (Fig. 2A), we further determined if other signaling events associated with autophagy induction were also modulated by adipoR2. As shown in Fig. 7, silencing of adipoR2 significantly suppressed gAcrp-induced increases in p21 (Fig. 7A), Nrf2 (Fig. 7B) and LC3II (Fig. 7C) expression in RAW 264.7 macrophages, whereas no significant effects were observed upon adipoR1 knockdown (only slight decrease in LC3II expression was observed), consistent with the results obtained from the regulation of p62 expression by adipoR2 (Fig. 2A). These results further confirmed that adiopoR2 would be the main receptor mediating gAcrp-induced p62 expression and autophagy activation in macrophages.

## Discussion

Adiponectin has been shown to possess potent anti-inflammatory properties through diverse mechanisms^44, 45^. Autophagy, a cellular self-digestive process, induces degradation of various target molecules, involving dysfunctional or damaged cellular components. There is a growing appreciation that autophagy plays a critical role in regulating the innate immune system and inflammatory responses^46^. In addition, a series of recent studies demonstrated the autophagy-inducing effects of adiponectin and its crucial role in modulating inflammatory responses^47^. While the potential role of autophagy in the management of inflammation has been well established, the underlying molecular mechanisms are still largely unknown. In the present study, we investigated the functional role of p62 in adiponectin-induced autophagy and modulation of inflammatory cytokine production in macrophages. Herein, we clearly demonstrated for the first time that p62 induction plays a vital role in the suppression of inflammatory cytokine production by globular adiponectin, at least in part, via autophagy induction. Moreover, we showed that the Nrf2/p21 axis is implicated in globular adiponectin-induced p62 expression.

p62 is implicated in various biological responses, including tumorigenesis, apoptosis, inflammation and autophagy^29, 48–50^. In particular, with regards to regulating autophagy, p62 delivers ubiquitin-bound protein complexes to the autophagosome and promotes degradation by autophagosome-lysosome fusion machinery. Meanwhile, p62 is also degraded along with the ubiquitinated substrates^26^. Therefore, cellular p62 level is speculated to be diminished during the process of autophagy. However, in the present study, we observed that globular adiponectin treatment induced a significant increase in p62 expression in macrophages (Fig. 1), although adiponectin induces autophagy under the same conditions. Additionally, p62 protein level was not further enhanced by Bafilomycin (Fig. 4A). Finally, gene silencing of p62 prevented gAcrp-induced increase in LC3II protein (Fig. 4B), collectively suggesting that p62 protein is not targeted for degradation by autophagy, but is implicated in autophagy induction. In fact, the biological roles of p62 in the regulation of autophagy are controversial. The results from the present study are indeed in accordance with recent studies demonstrating that p62 knockdown suppressed TLR4-mediated LC3 conversion in macrophages^51^, and attenuated autophagy leading to the inhibition of colorectal cancer cell growth^52^, indicating that the involvement of p62 in autophagy induction.

To further elucidate regulation of p62 by autophagy flux, we analyzed p62 level by gAcrp treatment at different time duration in the absence or presence of Bafilomycin (Supplementary Figure 8). Globular adiponectin increased p62 expression at relatively longer time incubation, but gAcrp-induced p62 expression was not further enhanced by Bafilomycin treatment consistent with the results from Fig. 4A. If the autophagy flux degrades cargo together with p62, p62 level would be further enhanced by Bafilomycin treatment. These results therefore suggest that p62 level would not be regulated by autophagy flux in this condition.

p62 cellular level is regulated by cell type and context specific manner. Although these results are not compatible with autophagy flux-mediated p62 regulation, but are consistent with the previous studies demonstrating the up-regulation of p62 expression even in the strong autophagy flux condition and when autophagy flux is functioning. Based on previous reports and the results from the present study, the regulation of p62 and its involvement in autophagy process is quite complicated and it seems not correlate to the autophagy flux process in all cases. At this stage, we couldn’t thoroughly address the underlying mechanisms and further studies will provide further insights into the mechanisms.

It has been well demonstrated that autophagy dysfunction is associated with various pathophysiological states including cancer, neuro-degeneration and aging^53, 54^. Recently, autophagy has been shown to modulate host defense system and has received much attention as a vital regulator of inflammation. For example, autophagy contributes to the development of tolerance to inflammatory cytokines^23, 55^. In the present study, we showed that autophagy induction plays a role in the suppressive effects of adiponectin on LPS-stimulated p38 MAPK phosphorylation and TRAF6 expression (Supplementary Figure 4A and B), consistent with the results from p62 knockdown (Fig. 3E and F) and previous studies^23^. These results are in agreement with the previous observations that autophagy induction by nuclear dot protein-52 (NDP52), an autophagy adaptor protein, promotes degradation of TRIF and TRAF6 in macrophages stimulated with a TLR3 agonist^56^. Based on the results demonstrating the autophagy-inducing effect of p62 (Fig. 4B–D) and the suppressive effect of autophagy on TRAF6/p38 MAPK pathway (Supplementary Figure 4A and B), it is clear that p62 induction plays a crucial role in suppressing inflammatory cytokine production by gAcrp via autophagy activation and suppression of TRAF6/p38 MAPK pathway.

Herein, we demonstrated the role of p62 signaling in the suppression of inflammatory cytokines production by gAcrp. However, p62 is also well known to induce inflammatory responses as discussed earlier. To further elucidate the role of p62 in modulating inflammatory cytokines expression, we examined the role of p62 in LPS-induced inflammatory cytokines production and found that p62 knockdown significantly suppressed expression of TNF-α and IFN-β without substantial effect on IL-1β expression (Supplementary Figure 6), indicating the involvement of p62 signaling in LPS-stimulated inflammatory cytokines production in a gene selective manner. Indeed, these results are contrary to those obtained from Fig. 3, which demonstrate that p62 signaling is involved in the suppression of inflammatory cytokines production by gAcrp. Based on these results, it appears that the role of p62 in modulating inflammatory cytokine is quite complicated and would be depending on the experimental environments. We assume that these conflicting results would be due to the different experimental (treatment) condition. In LPS alone treatment, the cellular p62 level is enhanced by LPS and this directly contributes to inflammatory cytokines expression. However, in case of treatment with adiponectin and LPS, pretreatment with adiponectin before stimulation with LPS activates various signaling cascades and biological responses, in addition to p62 induction, which may suppress inflammatory cytokines expression. One of the plausible mechanisms would be autophagy induction. As autophagy induction is known to elicit suppression of inflammatory cytokines production, it is possible that p62 induction during pretreatment with adiponectin results in autophagy induction finally leading to the inhibition of inflammatory cytokines expression. This hypothesis remains to be further investigated.

In addition, we also examined the role of p62 signaling in LPS-stimulated TRAF/p38MAPK axis. We observed that p62 gene silencing did not suppress TRAF6 expression (Supplementary Figure 7A), indicating that p62 signaling is not implicated in LPS-induced TRAF6 expression. Based on the previous report showing that p62 signaling induces TRAF6 ubiquitination, it appears that p62 controls activity of TRAF6 via (poly)ubiquitination, rather than modulating TRAF6 expression. In contrast to TRAF6 expression, p62 gene silencing significantly suppressed LPS-stimulated p38MAPK phosphorylation. Based on the previous reports showing that p38MAPK is a downstream target of TRAF6 and p62 induces TRAF6 ubiquitination, it is likely that p62 induction by LPS leads to p38MAPK phosphorylation through activation (ubiquitination) of TRAF6.

In addition to the role of Nrf2 in cellular defense mechanism via up-regulation of anti-oxidant cytoprotective genes, recent studies have indicated that Nrf2 is involved in p62 induction and closely associated with carcinogenesis and regulation of autophagic process. Under resting conditions, Nrf2 binds with Keap1 at ETGE and DLG (amino acid sequences present in Neh2 domain of Nrf2) motifs, resulting in its inactivation, ubiquitination and degradation in the cytosol^57^. However, in response to the stress that causes conformational changes in Keap1, the inhibitory effect of Keap1 is alleviated, resulting in prevention of Nrf2 degradation, enhancing its nuclear accumulation and prolonged Nrf2 activation^35^. Active Nrf2 binds with the antioxidant response element of the p62 promoter region and initiates transcriptional activation^58^. In agreement with this notion, in this study, we observed that Nrf2 induction by gAcrp contributes to p62 mRNA expression (Fig. 5). On the other hand, interestingly, recent studies also demonstrated that p62 can directly interact with Keap1, leading to Keap1 sequestration into the autophagosome and dissociation of Nrf2 from Keap1, further preventing the ubiquitination and degradation of Nrf2^39^. In line with this, we also observed that p62 knockdown inhibited gAcrp-induced Nrf2 expression in macrophages (Supplementary Figure 5), suggesting a potential role for p62 signaling in gAcrp-induced Nrf2 expression. Taken together, these results indicate the mutual regulation between p62 and Nrf2 and generation of positive feedback loop in macrophages treated with adiponectin.

In addition to the up-regulation of p62, Nrf2 is known to induce transcriptional activation of many genes related with autophagy. For example, Nrf2 induction by cisplatin is linked to the increased expression of autophagy-related genes, including Atg3, Beclin1 and Atg12, which contributes to the resistance against cisplatin treatment in ovarian cancer cells^59^. In addition, expression of ULK1, Atg5, Atg7 and GabarapL1 was highly suppressed in Nrf2 knockout mice^60^. Thus, it is possible that Nrf2 signaling is involved in autophagy induction by gAcrp through transcriptional activation of other autophagy-related genes, as well as p62. Further studies would be required to understand the molecular mechanisms for the role of Nrf2 signaling in gAcrp-induced autophagy activation.

In the present study, adiponectin increased Nrf2 protein expression without significant effect on mRNA level (Fig. 5A and B), implying that Nrf2 is post-transcriptionally regulated by adiponectin. Of the various mechanisms for regulating Nrf2 protein expression, ubiquitination has been shown to play additional potential role in the maintenance of Nrf2 cellular level. Herein, we observed an inhibitory effect of gAcrp on ubiquitination-mediated Nrf2 degradation (Fig. 6D), which was abrogated by p21 knockdown (Fig. 6E), suggesting a crucial role for p21 in gAcrp-induced stabilization of Nrf2. While p21 is well known to regulate cell cycle, herein, we found that p21 induction is closely associated with adiponectin-induced autophagy activation via enhancing Nrf2 activity and p62 expression. In the modulation of Nrf2 activity, p21 competes with Keap1 to bind at DLG motif of Nrf2, which leads to the stabilization and nuclear translocation of Nrf2^39^. Treatment with p21 inducers or p21 overexpression in response to oxidative stress repressed ubiquitination of proliferating cell nuclear antigen (PCNA), which is involved in DNA repair mechanism^61^. Based on previous reports and data from the current study, we suggest that p21 induction by adiponectin plays an important role in autophagy induction via modulation of Nrf2 stabilization.

Indeed, the regulatory role of p21 in autophagy induction is highly complicated. Overexpression of p21 increased susceptibility to autophagy induction, promoting tumour growth in several fibroblast cell lines, while opposite effects were observed in breast cancer cells^37^. Moreover, p21 overexpression negatively regulates autophagy in fibroblasts treated with a ceramide analogue^62^, while p21 induction correlates with autophagy induction in lung cancer cells treated with resveratrol^63^, implying that modulation of autophagy induction by p21 signaling would be depending on cellular context and growth environment. LPS stimulation induces inflammatory responses in macrophages deficient in p21^38^, suggesting that p21 contributes to the maintenance of immune system and modulates inflammation. Given that p21 potentially possesses anti-inflammatory properties and globular adiponectin induces p21 expression in macrophages, p21 may be a promising novel molecule implicated in anti-inflammatory responses by adiponectin.

Biological responses downstream of adiponectin are elicited through its distinct receptors. Adiponectin receptor type 1 (adipoR1) and type 2 (adipoR2) generate the signaling mechanisms almost identical, including activation of AMPK and/or PPAR-α^40^. In this study, we found that knocking down of adipoR2 prominently inhibited adiponectin-induced expression of p62 (Fig. 2A), p21, Nrf2 and LC3II (Fig. 7), suggesting that adipoR2-mediated signaling plays a predominant role in autophagy activation and suppression of inflammatory cytokines production in macrophages. With regards to autophagy induction by adiponectin, we and others demonstrated that AMPK signaling is implicated in adiponectin-induced autophagy activation^47, 64^. Of the two main adiponectin receptors, adipoR1 is well known to induce AMPK activation, while adipoR2 signaling is implicated in PPAR-α activation^65^. Therefore, the predominant role of adipoR2 signalingversus adiopoR1 in autophagy induction observed in this study would not be compatible with the crucial role of adipoR1 in activation of AMPK signaling. However, in contrast to the critical role of adipoR1 in AMPK activation, other studies also demonstrated the involvement of adipoR2 in the activation of AMPK signaling^1, 66^. In addition, adipoR1 and R2 modulate biological responses either similarly or differently depending upon the cellular environment. It is also possible that adiponectin-induced p62 expression and autophagy activation is mediated through signaling mechanisms other than AMPK signaling. At this stage, we could not thoroughly address the relationship between adipoR2 and AMPK activation. Future studies should provide further insights into the mechanisms required for understanding the role of adipoR2 in AMPK activation and modulation of autophagy in macrophages.

**Figure 8:**
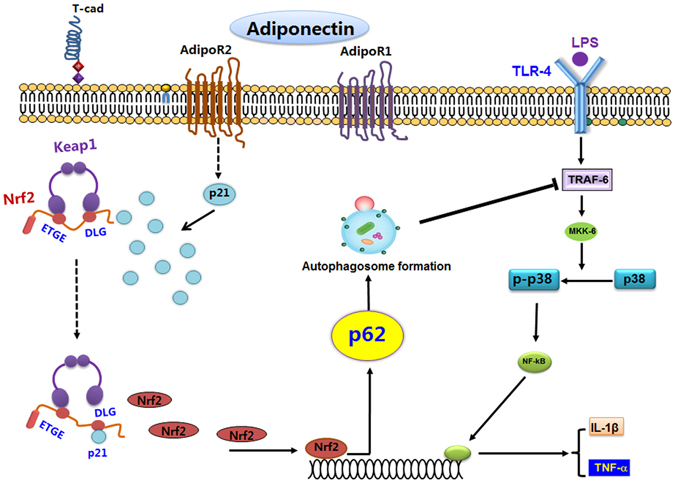
Proposed model for the suppression of inflammatory cytokine production by globular adiponectin in LPS-stimulated macrophages. LPS stimulates the production of various inflammatory cytokines including TNF-α and IL-1β through activation of TRAF6/p38 MAPK/NF-κB pathway in macrophages. Stimulation of macrophages with globular adiponectin results in the inhibition of LPS-stimulated inflammatory cytokines production via modulation of TRAF6 expression and p38 MAPK phosphorylation. The modulatory effects of globular adiponectin on inflammatory signaling cascade are mediated by autophagy induction. Increased p62 cellular level plays a vital role in globular adiponectin- mediated autophagy induction in macrophages, while the detailed mechanisms underlying autophagy activation by p62 remain to be investigated. Nrf2 plays a crucial role in transcriptional activation of p62 and p21 induction enhanced cellular Nrf2 level by preventing Nrf2 ubiquitination leading to its stabilization, collectively indicating that the p21/Nrf2 axis critically contributes to the up-regulation of p62 by globular adiponectin. Finally, it appears that anti-inflammation and autophagy inducing effects of globular adiponectin in macrophages are mainly generated through adipoR2-mediated signaling, rather than adipoR1. AdipoR1: adiponectin receptor type 1, AdipoR2: adiponectin receptor type 2, Keap1: Kelch ECH-associating protein 1, Nrf2: nuclear factor erythroid E2-related factor 2, TLR4: Toll-like receptor 4, TRAF6: TNF receptor-associated factor 6, ETGE/ DLG: amino acid sequence in Neh2 domain of Nrf2.

In conclusion, the data presented here indicate that globular adiponectin inhibits LPS-stimulated inflammatory cytokine expression, at least in part, via p62 induction and autophagy activation (Fig. 8). We demonstrated for the first time that p62 induction crucially contributes to the suppression of inflammatory cytokines production by globular adiponectin in macrophages through modulating the TRAF6/p38 MAPK pathway, which is triggered by autophagy induction. We also showed that Nrf2 induction was required for transcriptional activation of p62 by globular adiponectin. Furthermore, globular adiponectin enhanced cellular Nrf2 levels via prevention of Nrf2 ubiquitination and degradation through a p21-dependent mechanism. Based on these results, we propose that p62 induction mediated by the p21/Nrf2 axis is a novel signaling mechanism for autophagy induction and anti-inflammatory responses by adiponectin.

## Materials and Methods

### Materials

All cell culture reagents used in this study were obtained from HyClone Laboratories (South Logan, UT, USA). Recombinant human globular adiponectin (gAcrp) was purchased from Peprotech Inc. (Rocky Hill, NJ, USA). Bafilomycin A1, an inhibitor of autophagosome-lysosome fusion, was ordered from Sigma-Aldrich (St. Louis, MO, USA). Antibodies targeting p62 (Catalog. No. 5114), phospho-specific p38 MAPK (Catalog. No. 9215), total p38 MAPK (Catalog. No. 9212), LC3II (Catalog. No. 2775), Beclin-1(Catalog. No. 3738), and Nrf2 (Catalog. No. 8882) were purchased from Cell Signaling Technology Inc. (Beverly, MA, USA). Antibodies against TRAF6 (Catalog. No. Sc-7221), p21 (Catalog. No. sc-397) and ubiquitin (Catalog. No. sc-8017) were taken from Santa Cruz (Delaware, CA). Antibodies against Atg5 (Catalog. No. PAI-46178), β-actin (Catalog. No. 04-1116) and chemiluminescent substrate (product no. NCI4080KR) were from Thermo Scientific Inc. (Rockford, IL, USA). The TNF-α ELISA kits were purchased from BioLegend (San Diego, CA, USA). Until and unless mentioned elsewhere, all other chemicals were obtained from Sigma-Aldrich (St. Louis, MO, USA).

### Cell cultures

RAW 264.7 macrophage cell line (Catalog. No. KCLB-40071) was procured from Korean Cell Line Bank (Seoul, Korea) and was cultured in Dulbecco's modified Eagle medium (DMEM), supplemented with 10% (v/v) Fetal Bovine Serum (FBS) and 1% (v/v) penicillin-streptomycin. Cells were maintained at 37 °C in 5% CO_2_ and were sub-cultured every two days by splitting at a 1:10 ratio.

### Isolation and subculture of murine peritoneal macrophages

Animal experiments were performed following the guidelines issued by Yeungnam University Research Committee for proper care and handling of laboratory animals (YU-2016-009). The experimental protocols were approved by YU institutional animal care and use committee (IACUC). Peritoneal macrophages were isolated from 7- to 8-week old, male BALB/c mice essentially as described previously. Briefly, 4% (w/v) Brewer thioglycollate medium was injected intraperitoneal (i.p.) to stimulate macrophage accumulation in the peritoneal region. On third day after injection, peritoneal macrophages were extracted with ice-cold Hank's balanced salt solution (HBSS)-devoid of calcium and magnesium. The extracted cell suspension was then centrifuged at 300 × *g* for 5 min. Red blood cells were removed by lysing cells with RBC lysis buffer. Cells were then mixed in RPMI 1640 media containing 10% Fetal Calf Serum (FCS), 1% penicillin-streptomycin, and then seeded into culture dishes at a density of 1.5 × 10^6^  and incubated at 37 °C in an incubator with a humidified atmosphere containing 5% CO_2_ for further experiments.

### ELISA for TNF-α detection

5 × 10^4^ cells of RAW 264.7 macrophages were seeded in each well of 96-well plates and incubated for overnight. Cells were then transfected either with p62 siRNA (25 nM) or scrambled siRNA (25 nM). After 24 h of transfection, cells were treated with adiponectin for 24 h, followed by LPS treatment for an additional 4 h. The media were then collected and subjected to ELISA to detect secreted TNF-α protein using a TNF-α ELISA kit (BioLegend, San Diego, CA, USA) based on manufacturer's instructions.

### RNA isolation and Quantitative Real Time PCR (qRT-PCR)

To measure the mRNA levels of target genes, total RNA was isolated from cells by Qiagen lysis solution (Qiagen, Maryland, USA) and transcribed into cDNA by Go Script reverse transcription process (Promega) according to the manufacturer's instructions and as described previously^67^. Quantitative real time-PCR was then performed using a Light Cycler 2.0 (Mannheim, Germany) using absolute QPCR SYBR green capillary mix system (Thermo Scientific, UK) at 95 °C for 15 min, with successive 40 cycles of 95 °C for 15 s, 56 °C for 30 s, and 72 °C for 45 s. The mRNA level of target gene was normalized to the value of glyceraldehyde-3-phosphate dehydrogenase (GAPDH). The sequences of the primers used for the PCR amplification are listed in Table 1.

**Table 1:** Sequences of primers used in quantitative PCR.

Target gene	Primer	Nucleotide sequence
p62	F	5′-GCTCAGGAGGAGACGATGAC
	R	3′- AGAAACCCAAGGACAGCATC
TNF-α	F	5′-CCCTCACACTCAGATCATCTTCT-3′
	R	5′-GCTACGACGTGGGCTACAG-3′
GAPDH	F	5′-ACCACAGTCCATGCCATCAC-3′
	R	5′-TCCACCACCCTGTTGCTGTA-3′
IL-1β	F	5′-GCCTCGTGCTGTCGGACCCATAT
	R	3′-TCCTTTGAGGCCCAAGGCCACA
IL-6	F	5′-ACAACCACGGCCTTCCCTACTT
	R	3′-CACGATTTCCCAGAGAACATGTG
IFN-β	F	5′-AACTCCACCAGCAGACAGTG
	R	3′-TGAGGACATCTCCCACGTCA
Nrf2	F	5′-CTCGCTGGAAAAAGAAGTG
	R	3′-CCGTCCAGGAGTTCAGAGA
p21	F	5′-AATACCGTGGGTGTCAAAGC
	R	3′-GTGTGAGGACTCGGGACAAT
T-Cad	F	5′-TGCTGAAGACATGGCAGAAC
	R	3′-GGCTGACTCTGGTTCTCTGG

### Transient gene silencing by small interfering RNAs (siRNAs)

RAW 264.7 macrophages were seeded at a density of 7 × 10^5^ cells/dish in 35-mm dishes. Cells were transfected either with siRNA targeting specific genes or scrambled siRNA by means of Hiperfect transfection reagent (Qiagen) as directed by manufacturer. The transfection efficiency was measured by qRT-PCR or Western blot analysis 24 h after transfection. The siRNA duplexes were synthesized by Bioneer (Daejeon, South Korea) and sequences for the siRNA used are shown in Table 2.

**Table 2:** Sequences of small interfering RNA (siRNA).

Target gene	Primer	Nucleotide sequence
LC3B	F	5′-GUGGUUGUCAAGUGGUAGA-3′
	R	5′-UCUACCACUUGACAACCAC-3′
AdipoR1	F	5′-GACUUGGCUUGAGUGGUGU-3′
	R	5′-ACACCACUCAAGCCAAGUC-3′
AdipoR2	F	5′-AGAGUGAAGCCACCUGGUU-3′
	R	5′-AACCAGGUGGCUUCACUCU-3′
p62	F	5′-GCTCAGGAGGAGACGATGAC-3′
	R	5′-AGAAACCCATGGACAGCATC-3′
Nrf2	F	5′-GACUUUAGUCAGCGACAGA-3′
	R	5′-UCUGUCGCUGACUAAAGUC-3′
p21	F	5′-UGAGCAAUGGCUGAUCCUU-3′
	R	5′-AAGGAUCAGCCAUUGCUCA-3′

### Western blot analysis

Macrophages were seeded at a density of 1 × 10^6^ cells/dish in 35-mm dishes. After 24 h of incubation, cells were treated with globular adiponectin and/or other stimuli indicated. Total proteins were extracted using RIPA lysis buffer that contains phosphatase inhibitor single-use cocktail and proteases (Thermo Scientific, Waltham, MA). For immunoblot analysis, 20–30 μg of solubilized proteins were loaded and resolved by 8–15% SDS-PAGE. The proteins were transferred to PVDF membranes and nonspecific antigens were blocked with 5% skim milk prepared in phosphate-buffered saline/Tween 20 for 1 h. The membrane was then incubated with the appropriate primary antibody prepared in 3% bovine serum albumin (BSA) in shaker for overnight at 4 °C. The membrane was washed and incubated in the appropriate secondary anti-rabbit/mouse antibody tagged with horseradish peroxidase (HRP). The images of the blots were finally captured using a chemiluminescent substrate solution and Fujifilm LAS-4000 mini (Fujifilm, Tokyo, Japan).

### Preparation of conditioned media

Conditioned media were prepared as described previously^41^. After stimulation of macrophages with adiponectin for 8 h, culture media were removed and the cells were washed with warm PBS. Cells were then further incubated with fresh media (DMEM) for 12 h or 24 h. The media containing secreted mediators were then collected and centrifuged for 5 min at 5,000 × *g*. The supernatants were collected and mixed with fresh media at a ratio of 2:1 and were used to stimulate the new macrophages. Media collected from the cells untreated with adiponectin were used as an unstimulated control.

### Confocal microscopic analysis

RAW 264.7 macrophages were plated at a density 5 × 10^4^ cells/well in 8-well chamber slides and were allowed to adhere overnight. Cells were then transfected either with p62 siRNA or scrambled siRNA for 24 h, followed by overexpression of enhanced green fluorescent protein (eGFP)-LC3 plasmid with the help of Fugene HD transfection reagent (Promega, Madison, USA) for 48 h following the instructions given by manufacturer and as described previously^64^. After treatment with adiponectin for 24 h, cells were washed and fixed with paraformaldehyde solution. Finally, images were taken using A1 Confocal Laser Scanning Microscope System (Nikon Corp., Tokyo, Japan).

### Immunoprecipitation (IP) and immunoblot analysis

RAW 264.7 macrophages were plated in 100-mm dish at a density of 5.5 × 10^6^ cells/well and incubated overnight. Cells were then transfected either with siRNA targeting p21 or scrambled siRNA for 24 h, followed by stimulation with adiponectin for 6 h. Total protein was extracted with IP lysis buffer that contains 150 mM NaCl, 50 mM HEPES, 1% NP-40, 5 mM EDTA,1 mM PMSF and 0.5 mM DTT. 25 μL of Pierce Protein G Agarose (Thermo Scientific, Rockford, USA) was added to the lysates and incubated for overnight h at 4 °C with gentle rocking. Lysates were then centrifuged at 5,000 × *g* for 5 min and the supernatants were obtained. Five hundred µg of protein was incubated overnight with an anti-Nrf2 antibody diluted at a 1:50 ratio at 4 °C to form immune complexes. The complexes were then captured with 25 μL of protein G agarose for 4 h and the immune complexes were collected by centrifugation at 5,000 × *g* for 5 min. After washing with IP lysis buffer, the remaining beads were suspended in denaturation buffer and heated at 95 °C for 10 min. The denatured proteins were separated by SDS-PAGE electrophoresis, transferred onto PVDF membranes, and incubated with an anti-ubiquitin antibody. Proteins were visualized by chemiluminescent substrates as described earlier.

### Statistical analysis

Values were presented as mean ± S.E.M. from at least three independent experiments. Statistical significance was determined by ANOVA (one-way analysis of variance) and Tukey's multiple comparison using GraphPad Prism software.

## Electronic supplementary material

Supplementory information
