# Supplementary material for: Role of p62 in the suppression of inflammatory cytokine production by adiponectin in macrophages: Involvement of autophagy and p21/Nrf2 axis
Source: Sci Rep. 2017 Mar 24;7:393. doi: 10.1038/s41598-017-00456-6 (PMC5428427; doi:10.1038/s41598-017-00456-6)

Role of p62 in the suppression of inflammatory cytokine production by adiponectin in macrophages: Involvement of autophagy and p21/Nrf2 axis

Nirmala Tilija Pun[1] and Pil-Hoon Park[1]

[1]: College of Pharmacy, Yeungnam University, Gyeongsangbuk-do 712-749, Republic of Korea

Address correspondence to: Pil-Hoon Park, PhD

College of Pharmacy, Yeungnam University, Gyeongsangbuk-do 712-749, Republic of Korea.

Phone: 82-53-810-2826

Fax: 82-53-810-4654

Email: parkp@yu.ac.kr

Supplementary Figure 1. Effect of globular adiponectin on LPS-induced TNF-α mRNA expression in RAW 264.7 macrophages

RAW 264.7 macrophages were pretreated with gAcrp (0.5 μg/mL) for 24 h, followed by LPS (100 ng/ml) treatment for an additional 2 h. TNF-α mRNA expression level was measured by qRT-PCR. Values denote fold change relative to control and are presented as mean ± S.E.M. (n=3). *P < 0.05 compared to the control cells; $^{\#}$P < 0.05 compared to the cells treated with LPS.

Supplementary Figure 2. Effect of p62 siRNA on suppression of LPS-stimulated IFN-β mRNA expression by globular adiponectin in RAW 264.7 macrophages

RAW 264.7 macrophages were transfected with p62 siRNA or control siRNA. After 24 h of incubation, cells were then treated with adiponectin (0.5 μg/mL) for 24 h, followed by stimulation with LPS (100 ng/mL) for an additional 6 h. IFN-β mRNA level was evaluated by qRT-PCR and normalized with GAPDH mRNA. Values represent fold change relative to control and are presented as mean ± S.E.M. (n=3). *P < 0.05 compared to the control cells; $^{\#}$P < 0.05 compared to the cells treated with LPS.

Supplementary Figure 3. Effect of polymyxin B on regulation of TRAF6 expression and ERK phosphorylation by LPS and globular adiponectin in RAW 264.7 macrophages

Cells were pretreated with Polymyxin B (PMB) for 1 h, followed by LPS treatment for additional 30 min or cells were pretreated with Polymyxin B for 1 h, followed by

gAcrp for additional 24 h. The protein expression level of TRAF6 (A) and phospho-ERK (B) were detected by Western blot analyses. The representative of three independent experiments has been presented along with β-actin or total ERK as internal controls. Quantitative analyses of TRAF6 (A) and phospho-ERK (B) shown in lower panels were performed by densitometric analysis. Values are expressed as mean ± SEM (n = 3). *P < 0.05 compared to the control cells; #P < 0.05 compared to LPS treated cells.

Supplementary Figure 4. Effect of LC3 siRNA on suppression of LPS-stimulated p38MAPK phosphorylation and TRAF6 expression by adiponectin in RAW 264.7 macrophages

(A and B) Cells were transfected with LC3B siRNA or scrambled siRNA for 24 h. Cells were then treated with globular adiponectin for 24 h, followed by LPS treatment for additional 30 min. (Upper panel) Gene silencing efficiency of LC3B siRNA was monitored by Western blot analysis 24 h after transfection. Phosphorylated p38 MAPK (A) and TRAF6 expression (B) were detected by Western blot analysis along with internal control, β-actin. Images are the representative of three independent experiments. Quantitative analyses of the phosphorylated p38MAPK (A) and TRAF6 expression (B) by densitometric analysis are shown in the upper panels. Values are presented as mean ± SEM (n = 3). *P < 0.05 compared to the control cells; #P < 0.05 compared to the LPS treated cells; $P < 0.05 compared to the untransfected cells treated with LPS and adiponectin together.

Supplementary Figure 5. Effect of p62 siRNA on Nrf2 expression in RAW 264.7 macrophages

Cells were transfected either with p62 siRNA or scrambled control siRNA for 24 h and then further stimulated with globular adiponectin for additional 6 h. Total cellular extracts were prepared and used for the measurement of Nrf2 protein expression by Western blot analysis. Images are representative of two separate experiments. β-actin was indicated as an internal loading control. Quantitative analyses of Nrf2 shown in the lower panels were performed by densitometric analysis. Values are indicated as mean ± SEM (n = 2). *P < 0.05 compared to control; #P < 0.05 as compared to gAcrp treated groups.

Supplementary Figure 6. Effect of p62 siRNA on LPS-induced cytokines expression in RAW 264.7 macrophages

(A) Cells were transfected either with p62 siRNA or scrambled siRNA for 24 h and then stimulated with LPS (100 ng/ml) for 2 h (TNF-α mRNA) or 4 h (TNF-α protein secretion). The mRNA expression of TNF-α was measured by qRT-PCR (Upper panel) and secreted TNF-α level (Lower panel) was determined by ELISA. (B and C) Cells transfected with p62 siRNA or scrambled siRNA for 24 h and then stimulated with LPS (100 ng/ml) for 6 h. Messenger RNA expression level of IL-1β (B) and IFN-β (C) was measured by qRT-PCR. Values denote fold change relative to LPS and are presented as mean ± S.E.M. (n=4). *P < 0.05 compared to the control cells; $^{\#}P < 0.05$ compared to the cells treated with LPS and $^{\$}P < 0.05$ compared to the cells transfected with p62 siRNA and LPS treatment.

Supplementary Figure 7. Effect of p62 siRNA on LPS-induced TRAF6 expression and p38MAPK phosphorylatin in RAW 264.7 macrophages

Cells were transfected either with p62 siRNA or with scrambled siRNA for 24 h and then further stimulated with LPS (100 ng/ml) for 30 min and the expression of TRAF6 (A) and phospho-p38MAPK (B) were measured by Western blot analysis keeping β-actin or total p38MAPK protein as internal loading control. Images are the representative of three sets of independent experiments.

Supplementary Figure 8. Effect of Bafilomycin on the expression of LC3II and p62 protein by gAcrp in RAW 264.7 macrophages

Cells were pretreated with Bafilomycin A1 (10 nM) for 2h, followed by gAcrp treatment for different time durations. The expression of LC3II and p62 proteins were detected by Western blot analysis and presented a representative image from three sets of independent experiments. β-actin is an internal loading control.

Supplementary figure1

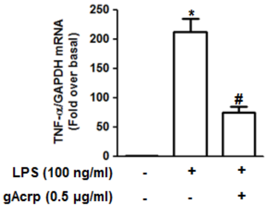

Supplementary figure 2

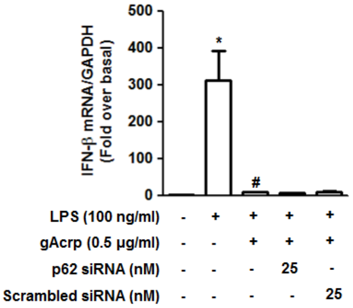

Supplementary figure 3

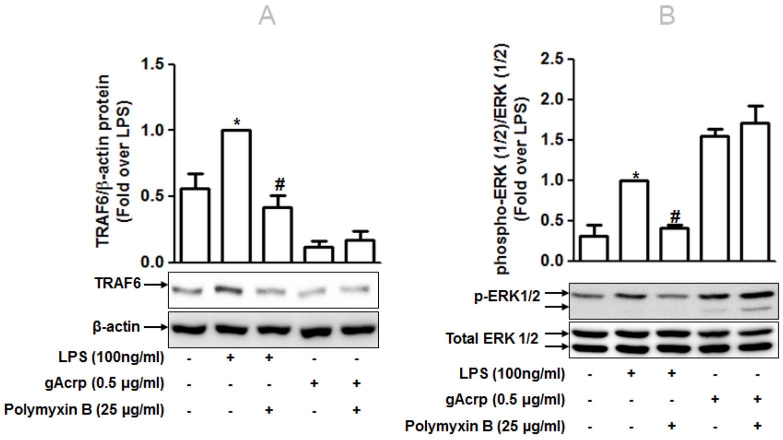

Supplementary figure 4

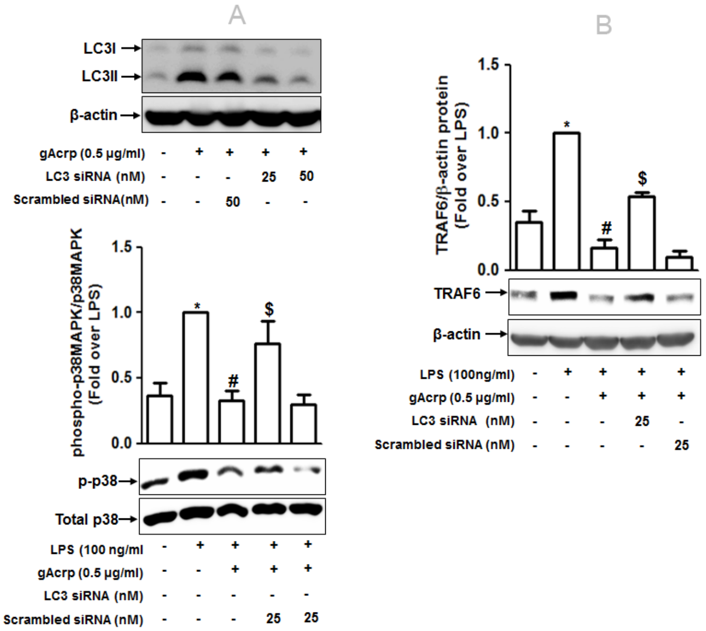

Supplementary figure 5

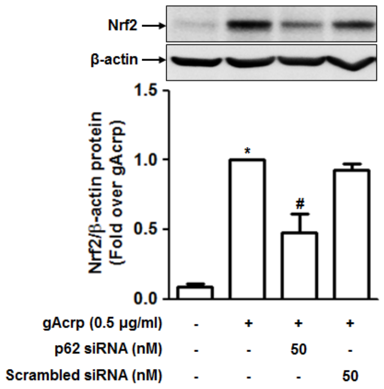

Supplementary figure 6

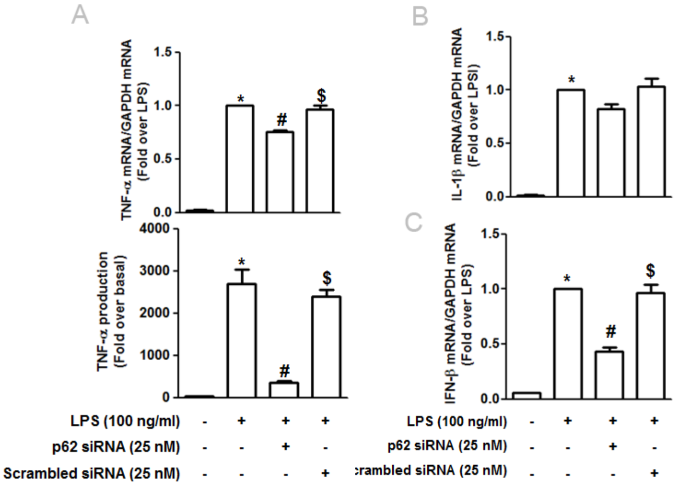

Supplementary figure 7

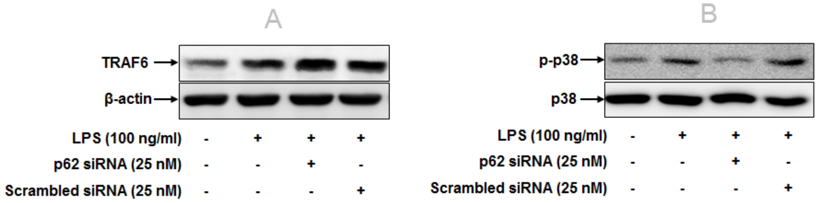

Supplementary figure 8

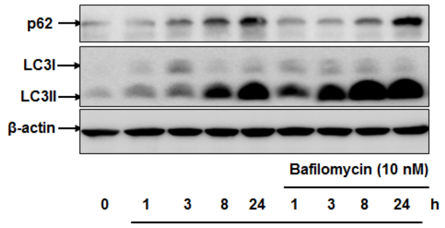

Supplement: Supplementary file 1 — Supplementory information [file 41598_2017_456_MOESM1_ESM.pdf]
